# Effects of Multiple Welding Thermal Cycles on Stress Corrosion of L360N Steel in a Simulated Shale Gas Gathering Environment Containing Sulfate-Reducing Bacteria

**DOI:** 10.3390/ma18184255

**Published:** 2025-09-11

**Authors:** Jie Xiao, Shouxi Wang, Yong Xu, Kexi Liao, Guiyang Wu, Jing Yan, Yongbo Wang, Lincai Peng, Puzhi Li

**Affiliations:** 1 College of Petroleum Engineering, Southwest Petroleum University, Chengdu 610500, China; 2 Research Institute of Natural Gas Technology, Petrochina Southwest Oil & Gasfield Company, Chengdu 610213, China; 3 College of Petroleum Engineering, Xi’an Shiyou University, Xi’an 710065, China; 4 PetroChina Southwest Oil and Gasfield Company, Chengdu 610051, China; 5 Southern Sichuan Gas District, Petrochina Southwest Oil & Gasfield Company, Luzhou 646099, China

**Keywords:** pipeline steel, welding, microbiologically influenced corrosion, sulfate-reducing bacteria, stress

## Abstract

The combined effect of sulfate-reducing bacteria (SRB) and a microstructure on the stress corrosion behavior of heat-affected zones (HAZs) in pipeline steel for shale gas field applications was investigated. The results show that when the peak heating temperature reached 1020 °C, a coarse microstructure formed during multiple thermal cycles (MTCs), and Widmanstätten structures appeared in the HAZ. In the simulated environment, SRB intensified localized pitting corrosion of both the base metal and the HAZ. The welding HAZ was softened by the MTCs, and significant microcrack growth was observed in the presence of SRB. Among all subzones, the coarse-grained HAZ (CGHAZ) was the most susceptible to stress corrosion cracking (SCC) under shale gas service conditions. Cracks initiated at the metal surface and propagated vertically into the material. SRB activity further increased the SCC sensitivity of the CGHAZ.

## 1. Introduction

Pipelines are widely used for the gathering and transportation of shale gas due to their low cost and high efficiency. In recent years, with the increasing demand for energy, shale gas exploitation has gradually expanded. However, shale gas reservoirs are rich in microorganisms, which can induce microbiologically influenced corrosion (MIC) and significantly reduce the service life of pipeline steels. Among these microorganisms, sulfate-reducing bacteria (SRB) are widely regarded as the primary culprits responsible for corrosion processes [1,2,3].

Previous studies have confirmed that environmental microorganisms can adhere to steel surfaces and form biofilms. The metabolic activity of these microorganisms can significantly increase the corrosion rate and reduce the ductility of pipeline steels [4,5]. Experimental research has shown that microbial activity plays a key role in accelerating localized corrosion. Since stress corrosion cracking (SCC) often originates from the base of pits or localized dissolution zones, SRB may play a positive role in promoting SCC initiation and propagation. Microbiologically assisted corrosion (MAC), which combines microbial effects with mechanical degradation, has been extensively studied under laboratory conditions [6,7,8]. Wu et al. [9] demonstrated that pipeline steels exposed to SRB-inoculated media experienced a substantial loss in ductility compared to those exposed to sterile conditions.

In practical applications, pipelines are typically joined by multi-layer and multi-pass welding. Due to the resulting heterogeneous microstructures and residual stresses, welded joints are generally the most vulnerable areas for corrosion and cracking. The heat-affected zone (HAZ), formed by the thermal cycle during welding, exhibits significant microstructural changes that influence both toughness and SCC resistance. However, the narrow width of real-world HAZs makes it challenging to extract samples directly for testing. As a result, thermal simulation techniques are frequently used to replicate the microstructures of HAZ subzones. For example, Ramachandran et al. [10] reported that a high fraction of high-angle grain boundaries in acicular ferrite microstructures can hinder crack propagation. Ashari et al. [11] investigated the effect of weld heat input on the corrosion behavior of dissimilar welded pipeline steels and found that higher corrosion rates in the HAZ were associated with the formation of acicular ferrite and secondary phases during welding.

Despite these advances, corrosion and cracking behavior in different HAZ subzones still remain insufficiently understood. It has been noted that even within the same welded joint, certain subzones of the HAZ are more susceptible to MIC under SRB exposure [12]. The MAC behavior of HAZ is governed by a combination of microbial activity, microstructural features, and mechanical factors [10,13,14]. However, most existing studies treat the HAZ as a single entity, and little attention has been paid to the distinct behavior of its subzones under MIC conditions.

To address these gaps, the present study investigates the combined effects of SRB activity and microstructural variation on the stress corrosion behavior of L360N steel HAZ for shale gas field applications. A simulated service environment representative of shale gas pipeline conditions was used to evaluate the corrosion performance of different HAZ subzones under SRB influence.

## 2. Materials and Methods

### 2.1. Materials

L360N pipeline steel, widely used in shale gas fields, was selected for this study. Its chemical composition is consistent with those reported in the literature [15]. All corrosion tests were performed on L360N carbon steel specimens which were obtained from Shandong Yangxin Shengxin Technology Co., Ltd. (Shandong, Jinan, China). To observe and analyze the microstructure, metallographic specimens were prepared by sequential grinding using SiC abrasive papers of 120#, 240#, 400#, 600#, 800#, 1000#, and 2000# grit. The samples were then polished with diamond paste to obtain a mirror-like finish, followed by etching with a 4% nitric acid alcohol solution. After etching, the samples were rinsed with deionized water and dried using cold air. Microstructural observations were carried out using an optical metallographic microscope (Zeiss, Axio Observer Z1,Oberkochen, Germany).

**Figure 1:**
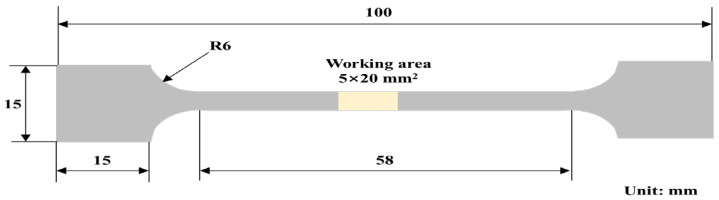
The dimensions of the tensile specimens stress corrosion test.

The dimensions of the samples used for stress corrosion testing are shown in Figure 1. For electrochemical measurements, samples were embedded in epoxy resin, leaving an exposed working area of 1 cm^2^. The exposed surfaces were polished sequentially with 120#, 240#, 400#, and 800# SiC papers, then rinsed with distilled water and anhydrous ethanol. Prior to testing, all specimens were sterilized under an ultraviolet (UV) lamp (Shanghai Boxun Medical Biotechnology Instrument Co., Ltd., Shanghai, China) for 30 min to eliminate surface microbial contamination.

### 2.2. Bacterial Cultivation and Inoculation

SRB (*Desulfovibrio desulfuricans*) were isolated from the flowback water of the shale gas field in Sichuan Basin was used in this work. The culturing medium was API RP-38 medium with a pH of 7.2, which contained the following (g/L): 1.0 ascorbic acid, 0.5 KH_2_PO_4_, 4.0 sodium lactate, 10.0 NaCl, 1.0 yeast extract, 0.2 MgSO4.7 H_2_O, and 0.02 Fe (NH_4_)_2_(SO_4_)_2_. Prior to inoculation, the culture medium was autoclaved for 20 min at 121 °C. And then the dissolved oxygen (DO) was removed by sparging the high-purity N_2_ for 1 h. In this work, an initial inoculum concentration of 10^6^ cfu/mL SRB was selected.

The growth and viability of *D*. *desulfuricans* in the test solution were quantified using the three-tube Most Probable Number (MPN) method, a standard technique for estimating viable anaerobic microbial populations. Measurements were taken at Day 0 and Day 14 to evaluate bacterial growth during the corrosion tests.

### 2.3. Test Solution

In this work, the simulated solution of shale gas fields was prepared as the test solution according to the laboratory analysis results. The simulated formation water composition was based on field sampling data from the target shale gas field in the Sichuan Basin. The chemical composition of the solution was shown in Table 1 with the CO_2_ content of 3%. The pH value was adjusted to 7.0 ± 0.2 using 1 mol/L HCl. Prior to the test, the solution was autoclaved for 20 min at 121 °C. The DO was also removed.

### 2.4. Welding Thermal Simulation

During the multi-pass welding process, different subzones within the HAZ experience distinct peak temperatures (T_p_) and cooling rates. The subzones affected by a single thermal cycle (STC) are typically classified as the coarse-grained HAZ (CGHAZ), fine-grained HAZ (FGHAZ), and intercritical HAZ (ICHAZ). In the case of multiple thermal cycles (MTCs), subsequent welding passes can further alter the microstructure of previously formed HAZs. The macrostructure of actual welded joints highlighting the HAZ regions and a schematic diagram of the welding thermal cycle curves can be referenced to the literature [16]. The thermal classification is based on the relationship between T_p_ and the critical transformation temperatures AC1 and AC3. Specifically, when T_p_ exceeds AC3 (the austenitization completion temperature), grain growth occurs, forming the CGHAZ. When T_p_ lies between AC3 and AC1 (the austenitization start temperature), partial transformation produces the ICHAZ. If T_p_ is slightly above AC3 without promoting significant grain growth, the FGHAZ forms. In this study, the welding thermal cycles were simulated using a Gleeble 3500 thermomechanical simulator (Dynamic Systems Inc., Albany, NY, USA) to reproduce the thermal history of each HAZ subzone. The target T_p_ values were determined based on measurements obtained during actual multi-pass GMAW using a high-resolution infrared thermal imaging device (Teledyne FLIR, Wilsonville, OR, USA). These representative peak temperatures were approximately 1350 °C (CGHAZ), 1100 °C (FGHAZ), and 800 °C (ICHAZ). During simulation, each specimen was equipped with a K-type thermocouple spot-welded near its center to ensure precise temperature monitoring and control. A consistent cooling time from 800 °C to 500 °C (t_8_⁄_5_) of 8 s was used to mimic typical thermal gradients observed in pipeline girth welds. Detailed simulation parameters are provided in Table 2.

### 2.5. Microstructure Analysis

The microstructures of the samples were first observed using an optical metallographic microscope. To further characterize the microstructural features, electron backscatter diffraction (EBSD) (Zeiss, Oberkochen, Germany) analysis was employed. This technique allowed for detailed analysis of the effects of multiple thermal cycles (MTCs) on the microstructural characteristics of the heat-affected zones (HAZs), including grain size, orientation, and grain boundary distribution.

Due to the sensitivity of EBSD, sample preparation followed a strict protocol. Specimens were first mechanically ground and polished, followed by electrolytic polishing using a solution of 20 vol.% perchloric acid in anhydrous ethanol. The applied voltage was set to 15 V, with the current controlled between 1.0 and 2.0 A depending on the immersion depth. Polishing was conducted for approximately 20 s under well-ventilated conditions at room temperature. EBSD data were collected using a ZEISS ULTRA 55 field-emission scanning electron microscope (Zeiss, Oberkochen, Germany) equipped with an HKL EBSD system. The acquired data were analyzed using HKL CHANNEL 5 software (2019V5.12).

### 2.6. Transmission Electron Microscopy (TEM)

Transmission electron microscopy (TEM) (Thermo Fisher Scientific, Waltham, MA, USA) was conducted to characterize the microstructural features of HAZs, including grain boundaries and dislocations. Samples were initially cut into 200 μm thick slices using a molybdenum wire cutting machine. These slices were then ground manually with sandpaper to a thickness of approximately 50–60 μm. Disks with a diameter of 3 mm were punched from the foils for final thinning. Double-jet electro-polishing was used to prepare the TEM foils, employing a solution of 5 vol.% perchloric acid in anhydrous ethanol cooled to −20 °C using liquid nitrogen. The TEM analysis was performed using a JEOL JEM-2100 transmission electron microscope (JEOL, Tokro, Japan) to observe and document fine microstructural features.

### 2.7. Mechanical Properties

Mechanical testing was carried out using a universal electronic testing machine (MTS, MN, USA)at a loading speed of 0.5 mm/min to determine tensile strength and cross-sectional reduction. Microhardness testing was conducted using a test load of 98 N. Hardness measurements were taken at multiple regions of each sample to evaluate the spatial distribution of hardness across different subzones.

### 2.8. Stress Corrosion Test

Stress corrosion testing was conducted using a custom-designed constant-load fixture equipped with a spring-loading mechanism. The tensile load was applied by adjusting the compression of the spring, and the corresponding pressure value was monitored in real time using an integrated electronic force gauge(Shanghai pavilion weighing instrument Co., LTD, Shanghai, China). The fixture ensured stable uniaxial loading without introducing bending or misalignment. A constant uniaxial tensile stress corresponding to 0.8σ0.2% proof strength of steel was applied during the immersion tests to simulate realistic service conditions in shale gas pipelines. Electrochemical impedance spectroscopy (EIS) and linear polarization resistance (LPR) tests were used to evaluate the effect of microstructure on the corrosion cracking behavior of welded joints in service environment. The frequency range measured by EIS was 100 kHz to 10 mHz with a voltage amplitude of 10 mV. The LPR measurement potential range was ±10 mV (vs. OCP) with a rate of 10 mV/min. Three parallel samples are used for testing.

## 3. Results and Discussion

### 3.1. Bacterial Growth Monitoring

The MPN results confirmed active growth of SRB during the 14-day immersion period. At the start of the experiment (Day 0), the SRB-inoculated solution contained approximately 10^6^ cells/mL, consistent with the initial inoculation target. After 2 days of anaerobic exposure, the SRB concentration increased to ~1.2 × 10^8^ cells/mL, indicating robust microbial activity. Subsequently, the number of cells gradually decreased, reaching 10^3^ on the 14th day. No bacterial growth was detected in the sterile control group, confirming the effectiveness of sterilization protocols.

### 3.2. Microstructure Analysis

**Figure 2:**
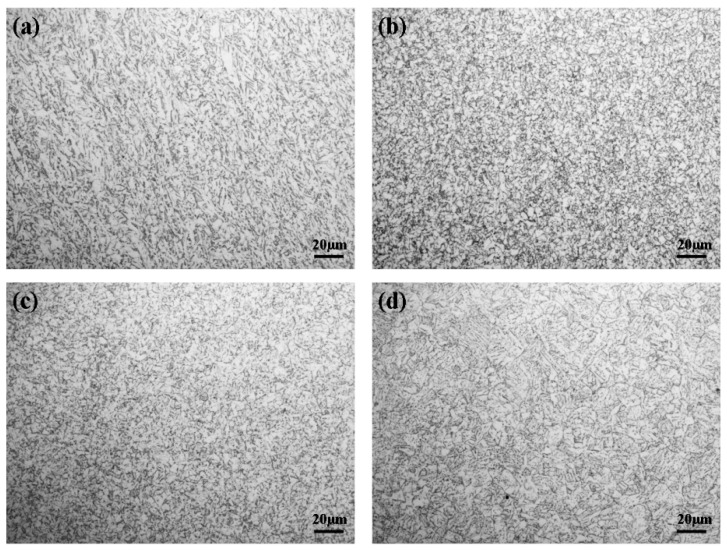
Metallographic structure of L360N pipeline steel: (**a**) BM; (**b**) 880 °C; (**c**) 950 °C; (**d**) 1020 °C.

To compare the microstructures of the typical subzones, the base metal (BM) of L360N pipeline steel is shown in Figure 2a. The BM microstructure consisted of ferrite and granular pearlite with a slight banded distribution, and the grains were relatively uniform in size. Figure 2b displays the intercritical grain heat-affected zone (IGHAZ), where a significant microstructural transformation was observed. The IGHAZ exhibited refined grain size and was primarily composed of fine granular bainite, polygonal ferrite, and M–A (martensite–austenite) islands.

The fine-grained heat-affected zone (FGHAZ), shown in Figure 2c, mainly consisted of granular bainite, polygonal ferrite, and M–A constituents, but the microstructure appeared less uniform in morphology and composition. In Figure 2d, the microstructure of the coarse-grained HAZ (CGHAZ) was dominated by coarse columnar ferrite with a small amount of pearlite. Additionally, Widmanstätten structures were observed, which are attributed to the high heat input during the welding thermal cycle, leading to grain coarsening and non-equilibrium transformations. These observations indicate that with increasing peak temperature during welding thermal cycles, the microstructure of the HAZ undergoes significant changes. The BM transitioned from a ferrite–pearlite structure to fine polygonal ferrite, and eventually to acicular or columnar ferrite in the CGHAZ. The M–A constituents also evolved—from isolated blocky forms to uneven distributions of blocky and strip-shaped features, and finally to elongated strips within the ferrite matrix. These microstructural transformations significantly influence the mechanical strength and stress corrosion cracking (SCC) susceptibility of the steel [17].

### 3.3. EBSD Results

**Figure 3:**
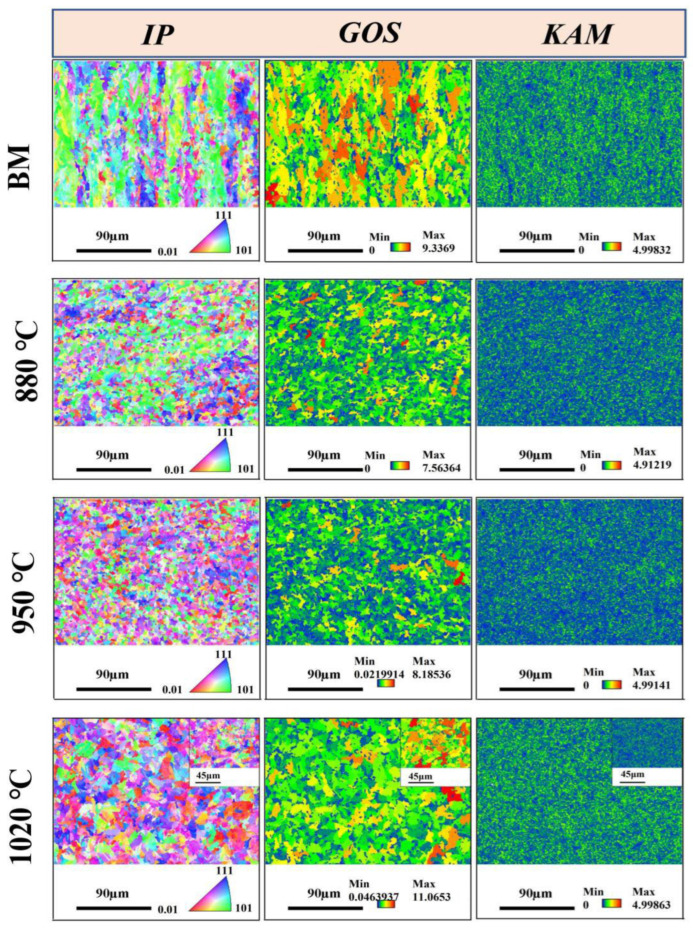
The inverse pole (IP), Grain Orientation Spread (GOS), and Kernel average misorientation (KAM) maps of L360N pipeline steel.

**Figure 4:**
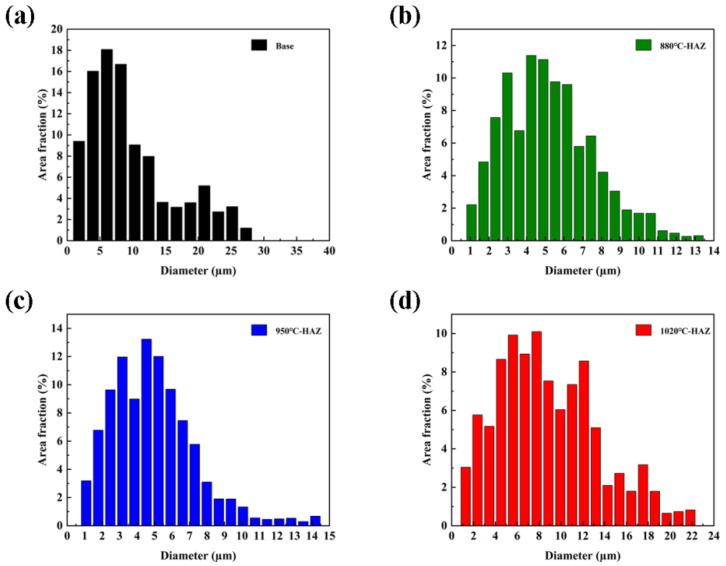
Statistical results of EBSD results: (**a**) base metal; (**b**) 880 °C; (**c**) 950 °C; (**d**) 1020 °C.

**Figure 5:**
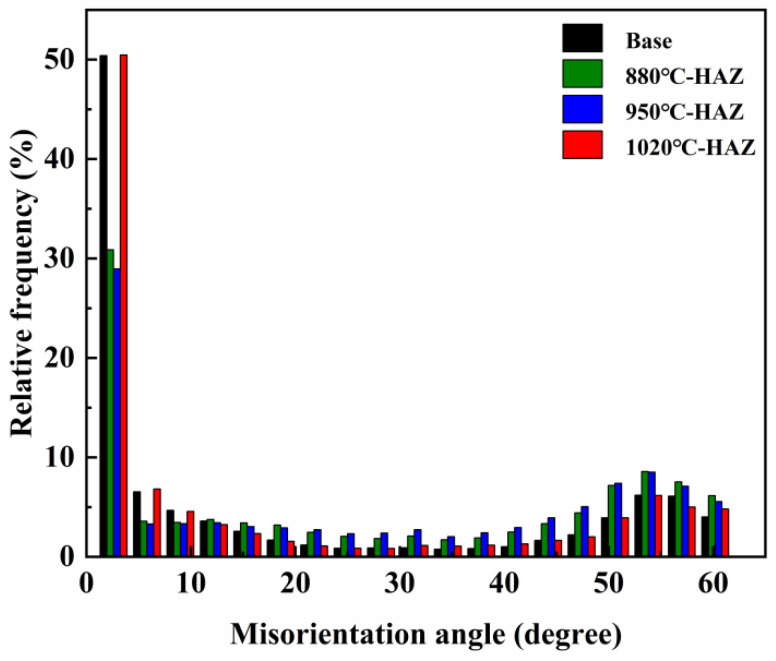
Statistical results of grain boundary angles.

The microstructures of the HAZs under different thermal conditions were characterized using EBSD, as shown in Figure 3. Statistical analysis of grain size, misorientation, and grain boundary characteristics is presented in Figure 4 and Figure 5. The IPF map of the BM displayed a pronounced parallel orientation band along the rolling direction, indicating a strong crystallographic texture. In contrast, the grain orientation in the IGHAZ appeared randomly distributed with no clear texture, suggesting significant microstructural transformation due to thermal cycling. The Grain Orientation Spread (GOS) maps reflected the level of elastic strain in the grains. Compared with the BM, the GOS peak values in the IGHAZ and FGHAZ were reduced, indicating lower residual strain in these zones. Conversely, the CGHAZ exhibited higher GOS values, suggesting that increased thermal cycling introduced greater residual stress. The KAM maps showed that the residual strain in the IGHAZ and FGHAZ was lower than that in the BM, further supporting the softening effect of thermal cycling in these subzones. In contrast, the CGHAZ showed elevated KAM values, implying higher residual stress levels. Grain size statistics revealed that the average grain size of the BM was approximately 9.7 μm, while the values for the IGHAZ, FGHAZ, and CGHAZ were 5.2 μm, 4.9 μm, and 8.8 μm, respectively. These results confirm that thermal cycling led to grain refinement in the lower peak temperature zones. Significant changes were also observed in grain boundary characteristics. The proportion of low-angle grain boundaries (<10°) decreased in the IGHAZ and FGHAZ, while it increased in the CGHAZ. Since a higher fraction of high-angle grain boundaries is generally associated with increased SCC susceptibility [18], these results suggest that microstructural evolution during welding can significantly influence localized corrosion behavior.

### 3.4. TEM Results

**Figure 6:**
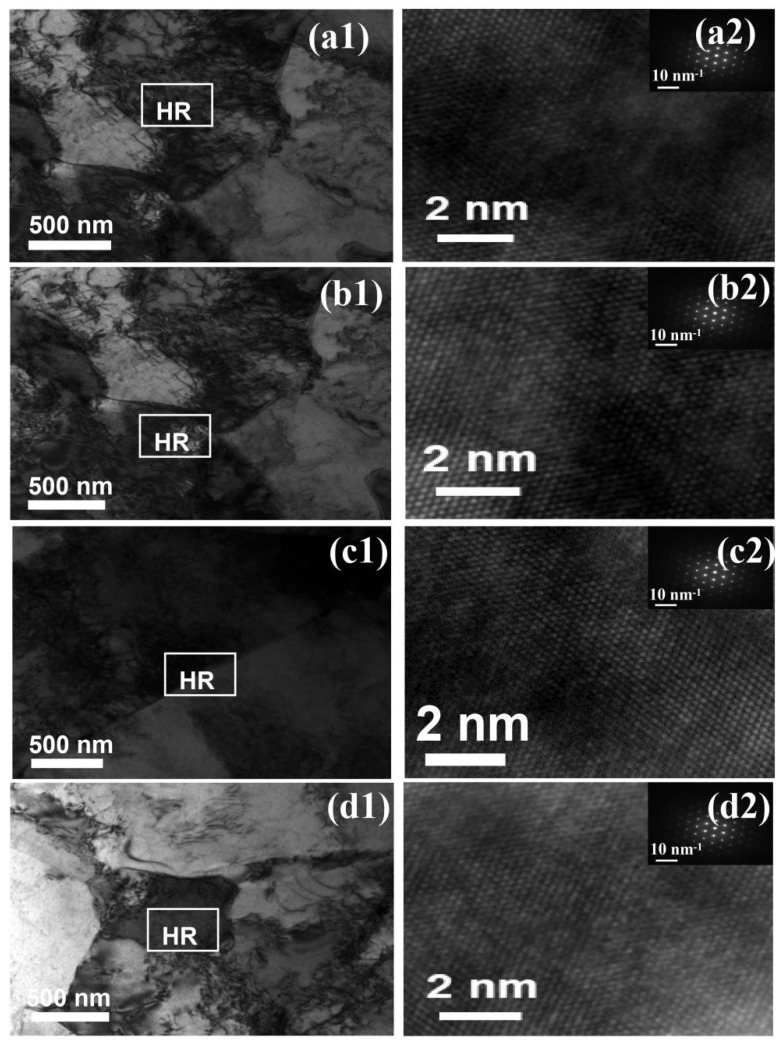
Typical TEM images and high-resolution characterization of L360N steel: (**a1**,**a2**) base metal; (**b1**,**b2**) 880 °C; (**c1**,**c2**) 950 °C; (**d1**,**d2**) 1020 °C.

TEM bright-field images of the HAZ samples and the corresponding selected area diffraction patterns (SADPs) are shown in Figure 6. In the hot-rolled state, the ferrite in the BM exhibited a relatively high dislocation density. High-resolution imaging further confirmed the presence of dislocation tangles, indicating prior deformation and strain hardening. The dislocation densities in the IGHAZ and FGHAZ were generally similar, with a marked increase in dislocation density both at grain boundaries and within ferrite grains. Dislocation accumulation along grain boundaries was evident, and characteristic dislocation cell and wall structures were observed, suggesting localized strain accommodation due to thermal cycling. In contrast, the CGHAZ showed a significant change in dislocation morphology. A noticeable reduction in dislocation density was observed compared to the other zones. This reduction is likely due to recrystallization or recovery processes associated with high heat input during welding. As reported in previous studies [19,20], such a decrease in dislocation density can contribute to microstructural softening, resulting in reduced strength and enhanced plasticity.

### 3.5. Tensile Properties

**Figure 7:**
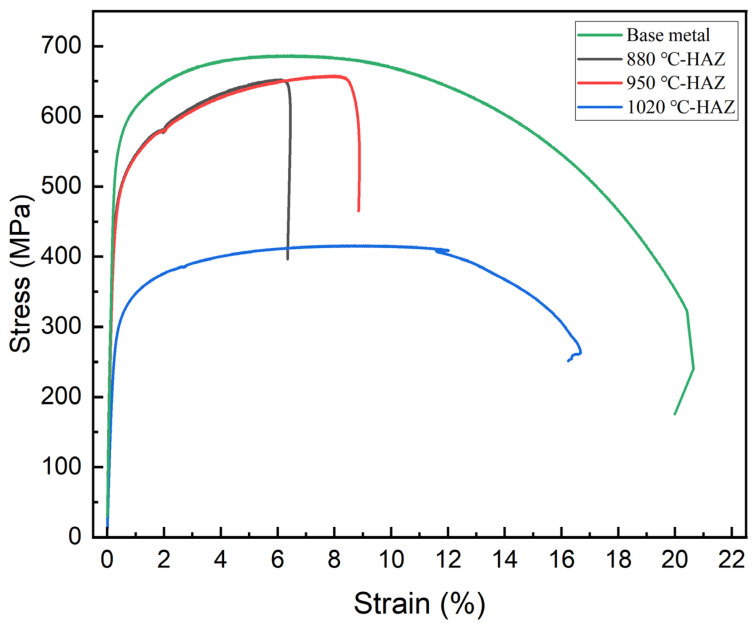
Engineering stress–strain curves for differently treated L360N steels.

The tensile properties of samples subjected to different thermal cycles are presented in Figure 7 and summarized in Table 3. It can be seen that both the yield strength and tensile strength of all HAZ samples were lower than those of the BM, indicating that welding thermal cycles led to a reduction in mechanical strength. Among the HAZ subzones, the CGHAZ exhibited the lowest strength values. As shown in Table 4, the yield strength and tensile strength of the IGHAZ were 495 MPa and 650 MPa, respectively. In the FGHAZ, these values slightly increased to 500 MPa and 655 MPa. However, when the peak temperature reached 1020 °C—representing the CGHAZ condition—the yield strength dropped significantly to 320 MPa, and the tensile strength declined to 413 MPa. These results clearly demonstrate that higher heat input during welding leads to pronounced softening and a substantial loss of mechanical performance in the HAZ.

### 3.6. Hardness Test

**Figure 8:**
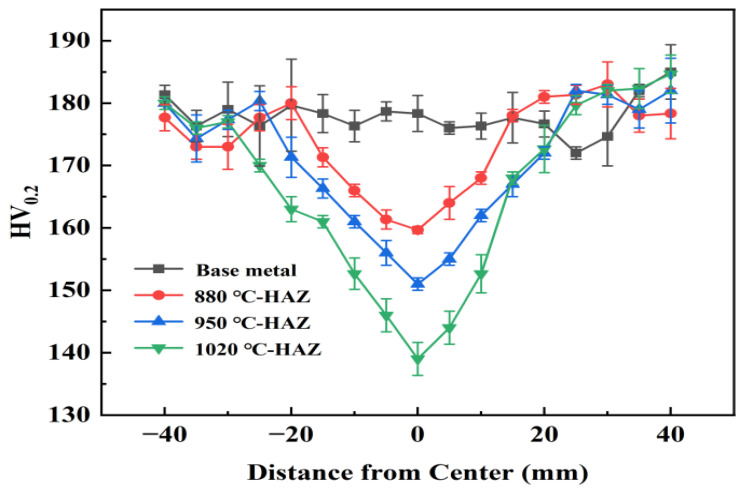
Hardness measurement results of differently treated L360N steels.

Figure 8 shows the microhardness distribution of the BM and various HAZ subzones in L360N pipeline steel. Compared with the BM, all HAZ regions exhibited significantly reduced hardness values. Among them, the CGHAZ had the lowest hardness, which can be attributed to the distinct thermal exposure in each subzone during welding. These temperature differences led to the formation of varying microstructures, thereby influencing hardness. The observed reduction in hardness, particularly in the CGHAZ, indicates a softening phenomenon caused by the effects of multiple thermal cycles (MTCs). This softening is consistent with the previously noted microstructural coarsening and reduction in dislocation density in this region.

### 3.7. Electrochemical Tests

#### 3.7.1. OCP and Linear Polarization Resistance (R_P_)

**Figure 9:**
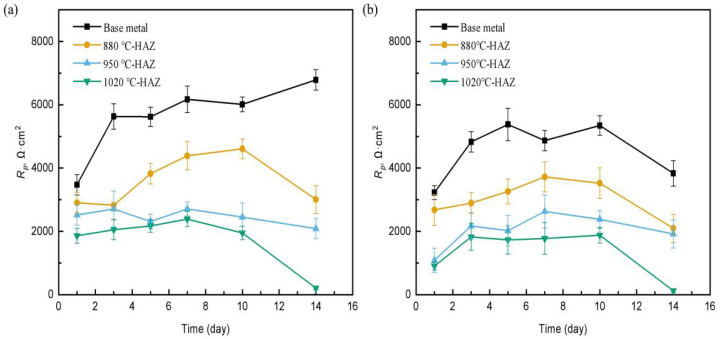
The R_p_ vs. time of differently treated L360N steels in (**a**) sterile and (**b**) SRB-inoculated simulated solutions.

The OCP values of all samples exhibited a gradual small increase (more positive) over time in the sterile medium. In contrast, the OCP in the SRB-inoculated solution became increasingly negative, especially in the CGHAZ and FGHAZ regions, reflecting enhanced electrochemical activity and the onset of localized corrosion under microbial influence (Table 4)*. R*_P_ can be used as a dynamic parameter to evaluate corrosion rate, and *R*_P_ is negatively correlated with corrosion rate. Figure 9 shows the *R*_P_ values of L360N steel and HAZs in the sterile and the SRB-inoculated simulated solutions for different times. In the absence of SRB, on the first day of the experiment, the *R*_P_ of the BM was 3473 Ω·cm^2^, the *R*_P_ values of the IGHAZ, FGHAZ, and CGHAZ samples were 2907 Ω·cm^2^, 2520 Ω·cm^2^, and 1862 Ω·cm^2^, respectively. The *R*_P_ of the CGHAZ sample was the smallest, indicating the highest corrosion rate. As the corrosion time increased, the *R*_P_ values of the BM gradually increased. On the 14th day of the test, the *R*_P_ value of the BM increased to 6700 Ω·cm^2^. This was because the formation of corrosion products on the steel surface played a protective role on the corrosion of the steel. Wei et al. [7] also reported that the formation of corrosion products increased the local potential. For the HAZs, the *R*_P_ values decreased on the 14th day. For the CGHAZ sample, the *R*_P_ decreased to 207.7 Ω·cm^2^. In the presence of SRB, the variation of *R*_P_ values showed a similar trend during the 14 days of corrosion. However, the *R*_P_ values of the BM and HAZs were lower than those of in the sterile solution. The above results indicate that the CGHAZ sample has a high stress corrosion sensitivity, and SRB enhanced the corrosion process.

#### 3.7.2. Electrochemical Impedance Spectroscopy (EIS)

**Figure 10:**
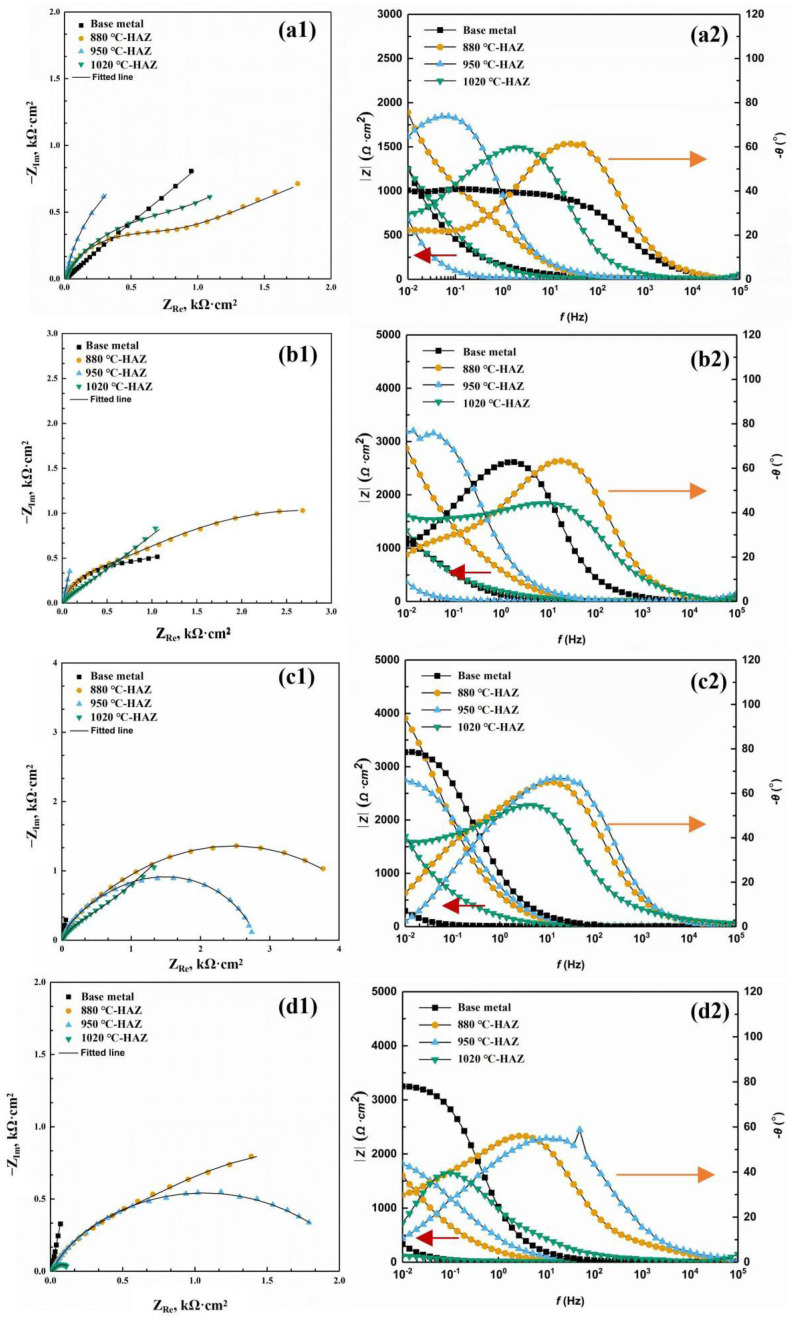
Nyquist and Bode plots for the steel during 14 days of testing in the sterile solution: (**a1**,**a2**) after 1 day; (**b1**,**b2**) after 7 days; (**c1**,**c2**) after 10 days; and (**d1**,**d2**) after 14 days.

**Figure 11:**
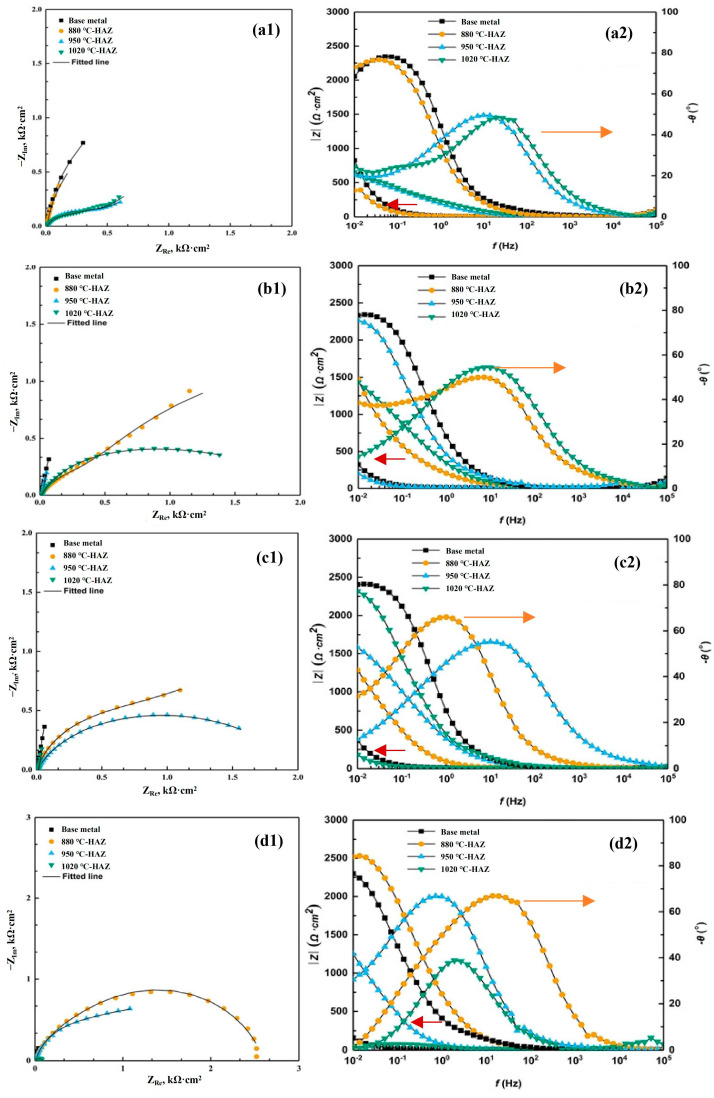
Nyquist and Bode plots for the steel during 14 days of testing in the SRB-inoculated solution: (**a1**,**a2**) after 1 day; (**b1**,**b2**) after 7 days; (**c1**,**c2**) after 10 days; and (**d1**,**d2**) after 14 days.

The EIS after different immersion times in a sterile environment and SRB-inoculated solutions was measured. The EIS results were shown in Figure 10 and Figure 11 in the form of Nyquist and Bode plots. In the absence of SRB, the capacitive arc radius of the BM was the largest, indicating that the corrosion rate of the BM was less than of the HAZs. Among the three types of HAZs, the radius of the capacitive arc of the CGHAZ sample was smaller than that of the IGHAZ and FGHAZ samples, indicating that the corrosion resistance of the CGHAZ sample was the lowest. In the presence of SRB, the similar characteristics can be observed.

In order to quantitatively analyze the EIS data, an equivalent circuit, *R*_s_ (*Q*_f_ (*R*_f_ (*Q*_dl_*R*_ct_)), was used to fit the obtained EIS spectrum. Replacing capacitive components with constant phase angle components, CPE can achieve better fitting results. *R*_s_ represents solution resistance, *Q*_f_ represents corrosion product capacitance, *R*_f_ represents corrosion product resistance, *R*_ct_ represents the electrical double layer resistance, and *Q*_dl_ represents the electrical double layer capacitance [21,22,23].

**Figure 12:**
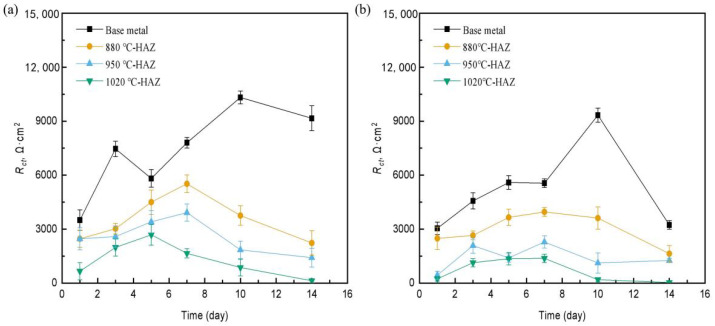
The Rct vs. time of differently treated L360N steels in (**a**) sterile and (**b**) SRB-inoculated simulated solutions.

Tables S1–S8 in the supplementary data show the EIS fitting results. *R*_ct_ can usually be used to evaluate the corrosion rate of steel. Figure 12 shows the *R*_ct_ values of L360N steel and HAZs in the sterile and the SRB-inoculated simulated solutions for different times. The *R*_ct_ values of the BM were all greater than those of the HAZs, indicating that the HAZs had the higher corrosion rate. Compared to the control (sterile condition), the *R*_ct_ values of the samples were relatively lower, which indicated that SRB enhanced the stress corrosion of steel. It is worth noting that a discrepancy was observed between the R_p_ obtained from LPR measurements and the *R*_ct_ derived from EIS under the same conditions. While *R*_ct_ reflects only the resistance associated with charge transfer at the metal–electrolyte interface, *R*_p_ from LPR encompasses both *R*_ct_ and additional resistances, such as the resistance of corrosion product films and potentially the biofilm layer in SRB-inoculated environments. Furthermore, the evolving surface conditions, including non-uniform corrosion and heterogeneous biofilm formation, can cause time-dependent variations in interfacial impedance and distort the ideal capacitive response. As a result, the time constants captured by LPR and EIS may not fully align, particularly in complex microbial systems. These factors collectively explain the variation between *R*_p_ and *R*_ct_ values in our experiments.

### 3.8. Characterization of Corrosion Products

**Figure 13:**
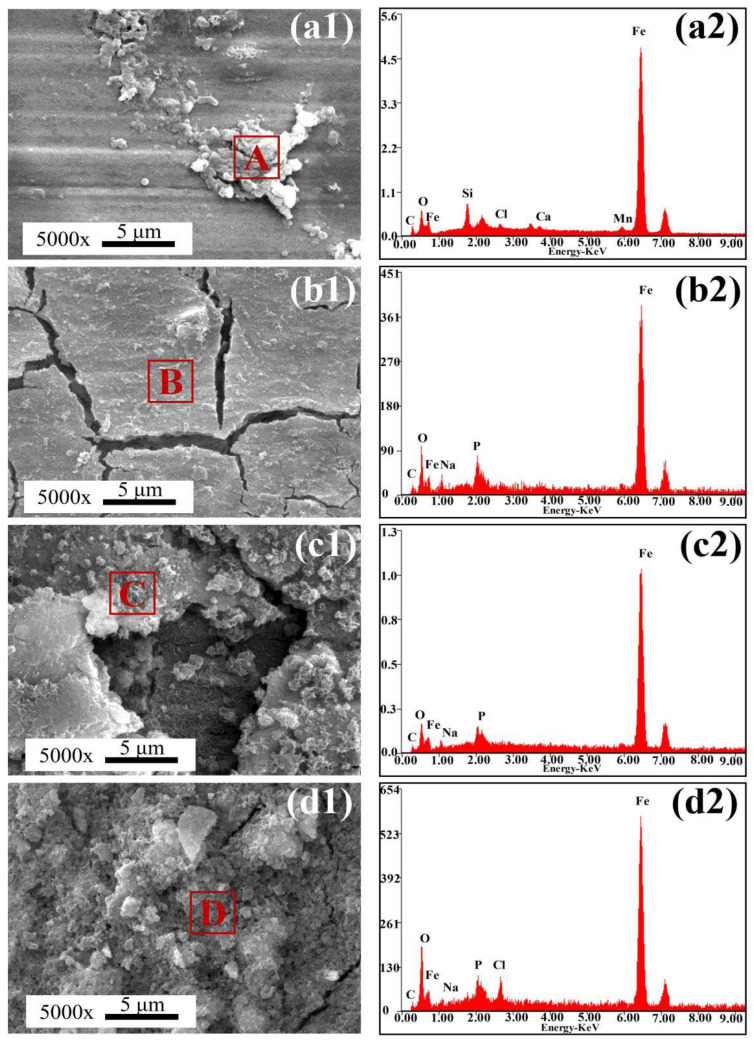
SEM images and EDS of surface corrosion products of samples after immersion in sterile simulated solution for 14 days: (**a1**,**a2**) BM; (**b1**,**b2**) IGHAZ; (**c1**,**c2**) FGHAZ; (**d1**,**d2**) CGHAZ, the letters “A–D” in subfigures (**a1**–**d1**) represent the EDS detection points.

Figure 13 shows the SEM images of corrosion products formed on the steel surfaces after 14 days of immersion in the sterile simulated solution. In the absence of SRB, only a small number of granular corrosion products were observed on the BM surface (Figure 13a1,a2). In contrast, the surfaces of all HAZ samples were completely covered with corrosion product films, indicating a higher corrosion tendency in these thermally affected regions. Elemental compositions of the corrosion products under sterile conditions, as shown in Table 5, mainly included C, O, Na, Si, Cl, and Fe, suggesting that the corrosion products were primarily iron oxides. In anaerobic environments, the corrosion process is governed by the oxidation of Fe and the reduction in H^+^, as described by the following electrochemical reactions [24]:

Anode reaction:

Fe^0^ → Fe^2+^ + 2e (1)

Cathode reaction:

H_2_O + e^−^ → H_ads_ + OH^−^ (2)

**Figure 14:**
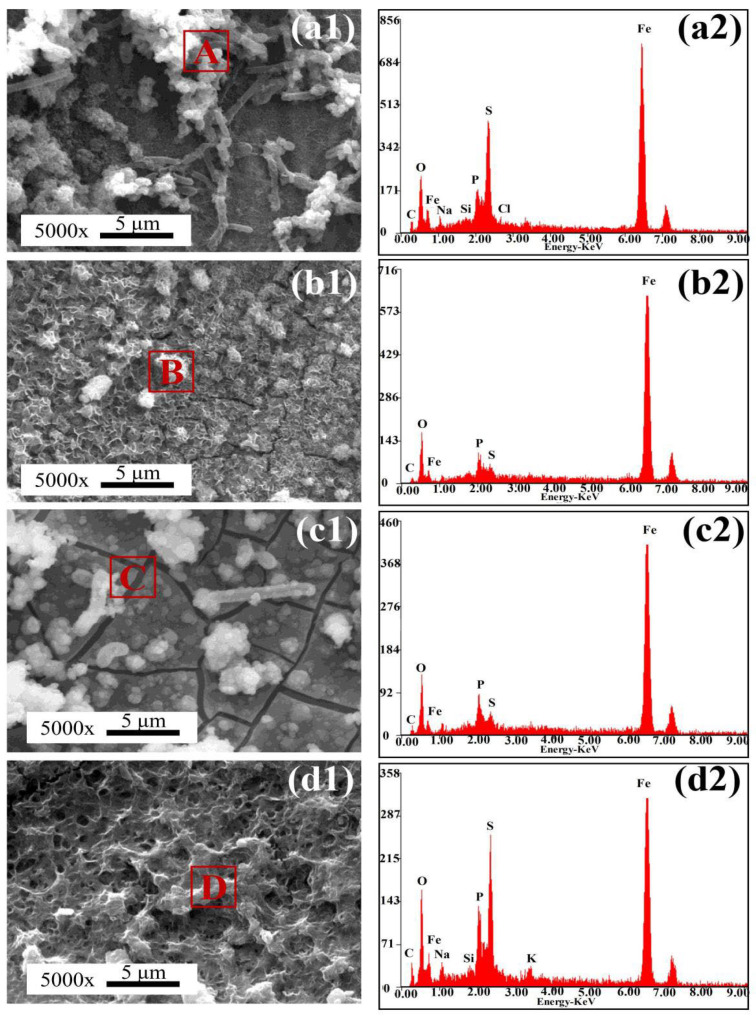
SEM images and EDS of surface corrosion products of samples after immersion in the SRB-inoculated simulated solution for 14 days: (**a1**,**a2**) BM; (**b1**,**b2**) IGHAZ; (**c1**,**c2**) FGHAZ; (**d1**,**d2**) CGHAZ, the letters “A–D” in subfigures (**a1**–**d1**) represent the EDS detection points.

Figure 14 shows SEM images of corrosion products on the steel surfaces after 14 days of immersion in the SRB-inoculated simulated solution. On the BM surface, only a small amount of clustered corrosion products was observed, along with the presence of numerous SRB cells. For the HAZ samples, the entire surface was covered with a dense corrosion product film embedded with SRB cells. In the IGHAZ, the corrosion film appeared as a clustered layer. In the FGHAZ, the film exhibited visible cracks. For the CGHAZ, the corrosion film was relatively intact, displaying typical biofilm morphology resulting from SRB metabolic activity.

EDS analysis results of the corrosion products in the SRB environment are summarized in Table 6. The products were mainly composed of C, O, Na, Si, Cl, S, and Fe, indicating that both iron oxides and iron sulfides were present. The detection of sulfur in the SRB group further confirmed SRB participation in the corrosion process. In SRB-induced corrosion, Fe^0^ in the steel matrix is oxidized extracellularly, while sulfate reduction occurs intracellularly via enzymatic catalysis. The electrons released during Fe oxidation are transferred into the SRB cytoplasm [24,25,26], where the following biocatalytic reaction occurs:

Biocatalytic cathodic sulfate reduction:

SO_4_^2−^ + 9 H^+^ + 8e → HS^−^ + 4H_2_O (3)

Fe^2+^ + HS^−^ → FeS + H^+^ (4)

### 3.9. Characterization of Morphology After Removal of the Corrosion Products

**Figure 15:**
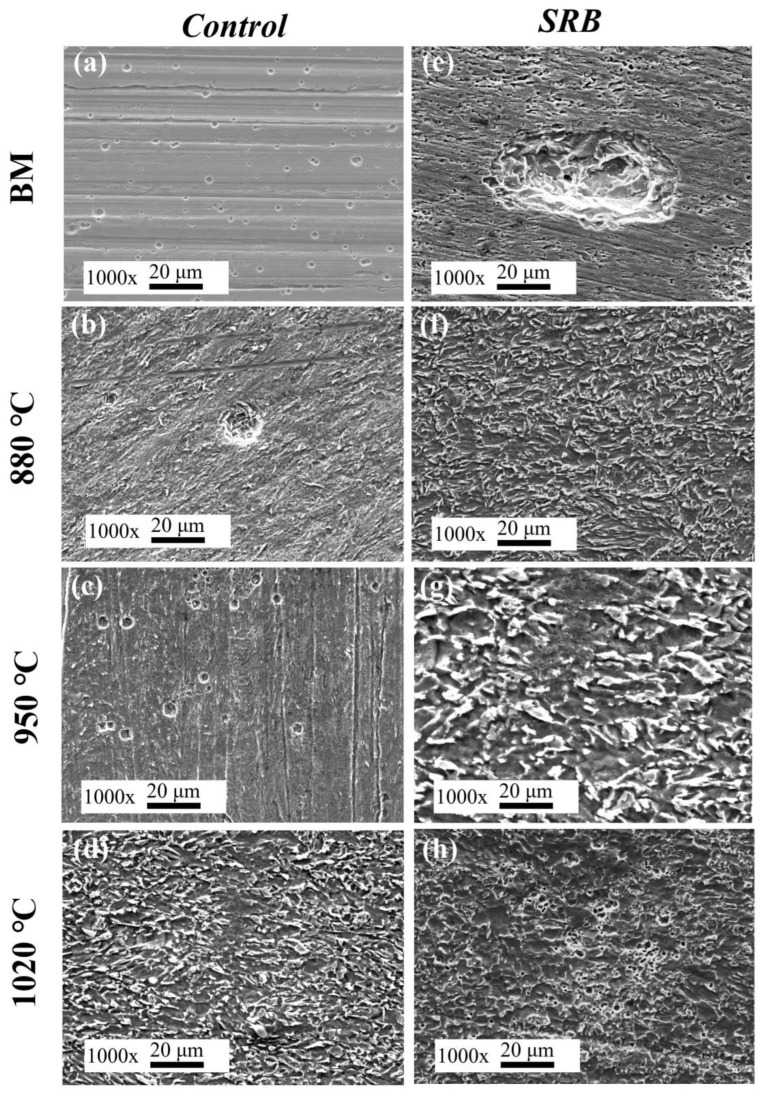
SEM images and EDS of surface corrosion products of samples after immersion in sterile simulated solution for 14 days: (**a**,**e**) BM; (**b**,**f**) IGHAZ; (**c**,**g**) FGHAZ; (**d**,**h**) CGHAZ.

Figure 15 shows SEM images of the steel surface morphology after 14 days of immersion in the simulated solution, following the removal of corrosion products. In the absence of SRB, only a few small pits were observed on the BM surface, indicating that L360N pipeline steel primarily experienced localized pitting corrosion. This type of corrosion is attributed to the presence of chloride ions (Cl^−^) in the environment.

In contrast, significant differences in corrosion morphology were observed among the HAZ samples. The IGHAZ exhibited a relatively uniform corrosion pattern, while the FGHAZ showed localized corrosion with the presence of visible pits. The CGHAZ surface displayed severe and widespread uniform corrosion. These findings highlight that the corrosion behavior of HAZs differs markedly from that of the BM due to the variations in microstructure induced by thermal cycles.

Under SRB-inoculated conditions, a large and deep pit was observed on the BM surface, confirming that SRB accelerated localized corrosion. For the HAZ samples, the corrosion morphology shifted to more uniform attack across the surface. This behavior is attributed to the formation of a dense biofilm, which alters the local microenvironment by creating ion concentration gradients, oxygen diffusion limitations, and redox heterogeneity across the metal–biofilm interface. These microenvironmental changes significantly influence the corrosion pattern and progression.

### 3.10. Pits Analysis

**Figure 16:**
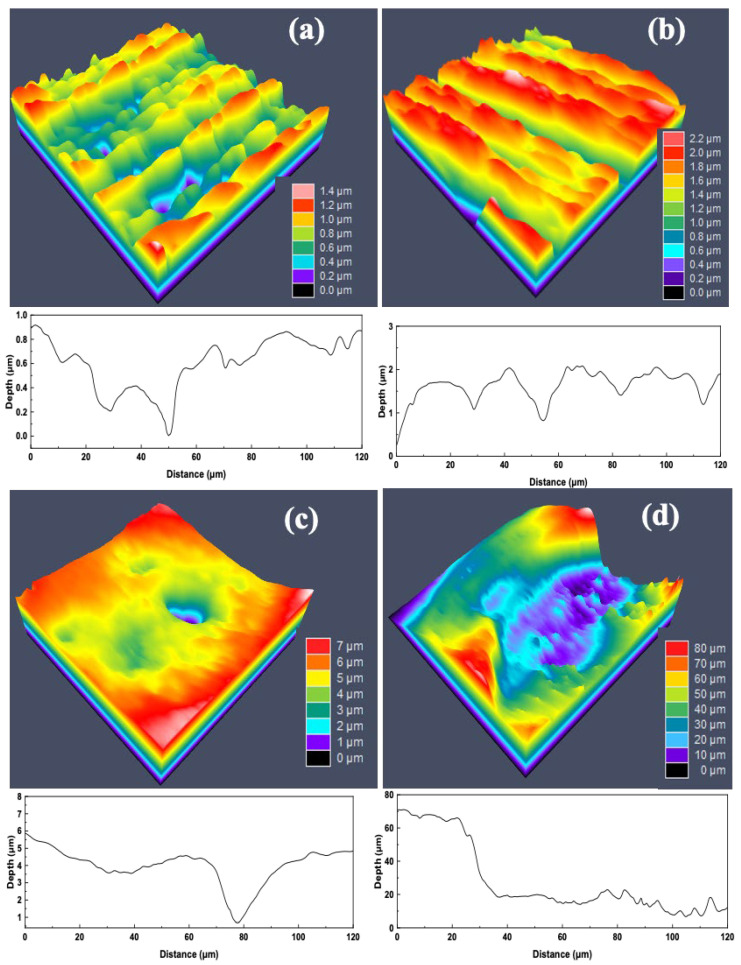
CLSM diagram of the largest pitting corrosion pit on the surface after immersion in the sterile simulated gas solution for 14 days: (**a**) base metal; (**b**) 880 °C; (**c**) 950 °C; (**d**) 1020 °C.

**Figure 17:**
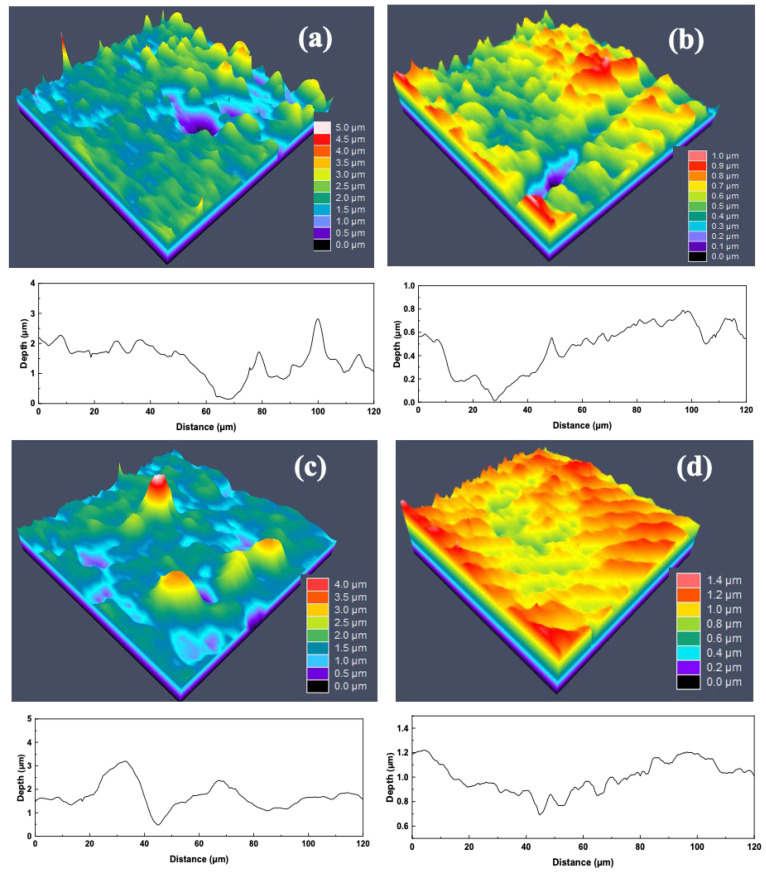
CLSM diagram of the largest pitting corrosion pit on the surface after immersion in the SRB-inoculated simulated gas solution for 14 days: (**a**) base metal; (**b**) 880 °C; (**c**) 950 °C; (**d**) 1020 °C.

To further investigate the pitting corrosion behavior of pipeline steel, the surface morphology of pits formed under different conditions was analyzed using laser scanning confocal microscopy (CLSM) [27]. Figure 16 and Figure 17 present CLSM images of the deepest pits observed on the surfaces of the BM and HAZ samples after 14 days of immersion in sterile and SRB-inoculated simulated solutions, respectively. In the sterile solution, the BM exhibited only shallow pitting, with the maximum pit depth around 1 μm. In the IGHAZ sample, the pit depth increased slightly to approximately 2 μm. The FGHAZ sample showed more pronounced localized corrosion, with pits reaching a depth of 62 μm. Interestingly, the CGHAZ exhibited a maximum pit depth of approximately 50 μm, indicating severe pitting even in the absence of SRB, likely due to microstructural softening and higher corrosion susceptibility. In the presence of SRB, the maximum pit depths for all samples increased compared to those in the sterile group, confirming that SRB significantly enhanced localized corrosion. The intensified pitting can be attributed to the metabolic activity of SRB and the associated formation of corrosive metabolites such as hydrogen sulfide, which further degrade the passive film and promote pit propagation.

### 3.11. Cross-Sectional Morphology

**Figure 18:**
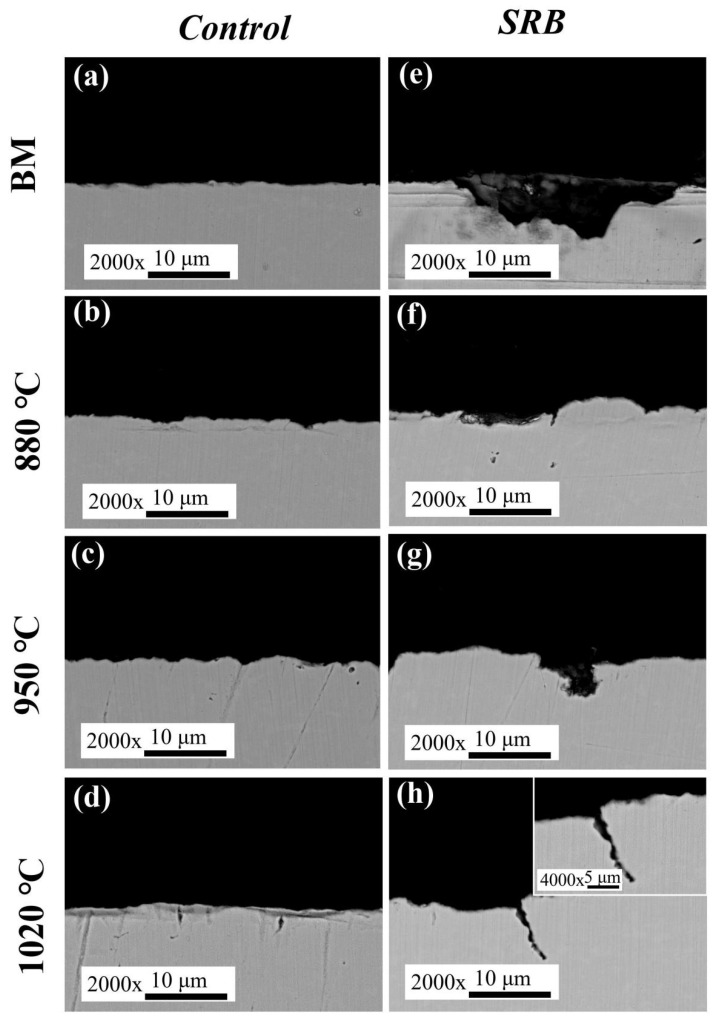
SEM sectional morphology of steel after 14 days immersion in the sterile and SRB-inoculated simulated solutions: (**a**,**e**) BM; (**b**,**f**) IGHAZ; (**c**,**g**) FGHAZ; (**d**,**h**) CGHAZ.

Figure 18 shows the cross-sectional morphologies of the steel samples after 14 days of immersion in the simulated solution. In the sterile environment, the BM surface remained smooth, and no visible cracks were observed (Figure 18a). Similar results were observed for the IGHAZ and FGHAZ samples (Figure 18b,c), where the surfaces showed no evidence of cracking. However, in the CGHAZ sample (Figure 18d), several microcracks were observed, indicating that this region is more susceptible to cracking, even in the absence of microbial influence. In the SRB-inoculated environment (Figure 18e–h), crack formation was significantly intensified in the CGHAZ. These cracks exhibited the following characteristics:

a. Crack initiation occurred at the metal surface, followed by vertical propagation into the material;
b. The cracks were long and narrow, with lengths exceeding 10 μm;
c. A characteristic “cloth-bag-shaped” morphology was observed.

These features are consistent with typical crack propagation behavior associated with microbiologically assisted cracking (MAC) under field conditions. The results indicate that the CGHAZ is particularly prone to microbiologically influenced stress corrosion cracking (MISCC) under simulated shale gas service environments. Furthermore, these findings demonstrate that the applied tensile stress enhances the mechanochemical activity of the steel. From a thermodynamic perspective, considering mechanochemical interactions, the electrochemical activity of a material under stress can be described by a shift in the equilibrium electrochemical potential (Δφ_0_), as shown in Equation (5) [28]:

(5) $$∆\varphi_{0}=-∆\mathrm{PVm}/\mathrm{zF}$$

Under applied tensile stress, the equilibrium potential of the steel shifts in the negative direction, thereby increasing its electrochemical reactivity. Consequently, the corrosion tendency is enhanced by mechanical loading. In the presence of SRB, pitting corrosion was the dominant degradation mode, consistent with prior studies [29,30,31,32,33]. In this work, the presence of surface pits was also confirmed. Under the combined influence of microbial activity and mechanical stress, cracks preferentially initiated at pit sites due to stress concentration effects. Therefore, the susceptibility to SCC increased significantly under the coupled effect of SRB and stress.

## 4. Conclusions

In this study, the combined effects of mechanical stress and sulfate-reducing bacteria (SRB) on the heat-affected zones (HAZs) of L360N pipeline steel, subjected to different heat inputs via Gleeble thermal simulation, were systematically investigated for shale gas field applications. The microstructure, hardness distribution, dislocation evolution, mechanical properties, and electrochemical corrosion behavior were comprehensively analyzed. The main conclusions are as follows:

1. Significant microstructural differences were observed between the base metal (BM) and various HAZ subzones. When the peak temperature (T_p_) reached 1020 °C, coarse microstructures and Widmanstätten structures formed in the HAZ due to high heat input. The dislocation structure evolved markedly, with a reduction in dislocation density and the disappearance of entanglement and accumulation. These changes led to microstructural softening, resulting in reduced strength and increased plasticity.
2. In the simulated environment, the corrosion behavior of the HAZ samples differed from that of the BM. The IGHAZ primarily exhibited uniform corrosion, while the FGHAZ showed pitting corrosion. The CGHAZ displayed the most severe corrosion in the form of widespread uniform attack and fine pits. In the presence of SRB, localized pitting corrosion was significantly intensified in both the BM and HAZs, with the CGHAZ showing the most pronounced degradation.
3. When the T_p_ reaches 1020 °C, the CGHAZ was softened by multiple thermal cycles (MTCs), and noticeable microcrack growth occurred in the simulated shale gas environment. This indicates that the CGHAZ is particularly susceptible to SCC under service conditions. In the presence of SRB, the crack morphology changed notably—cracks initiated at the metal surface and propagated vertically downward. SRB activity further increased the SCC sensitivity of the CGHAZ by promoting pit-induced crack initiation and propagation.

## Figures and Tables

**Table 1:** Chemical composition of shale gas quality simulation solution.

Chemical	NaHCO_3_	NaCl	CaCl_2_	Na_2_SO_4_	K_2_SO_4_	MgSO_4_
Concentration (mg L^−1^)	2900	20,000	395	260	170	50

**Table 2:** Process parameters used to simulate single welding thermal cycle.

No.	Heating Rate *ω*_H_/(°C·s^−1^)	Peak Temperature T_p_/°C	Holding Time *t*/s	Cooling Time *t*_8/5_/s	Cooling Time *t*_5/3_/s
1	160	880	1	8	30
2	160	950	1	8	30
3	160	1020	1	8	30

**Table 3:** Mechanical properties of differently treated L360N steels.

Samples	Yielding Strength σ_0.2_ (MPa)	Tensile Strength σ_b_ (MPa)	σ_0.2_/σ_b_
Base metal	540	680	0.79
IGHAZ	495	650	0.76
FGHAZ	500	655	0.76
CGHAZ	320	413	0.77

**Table 4:** OCP values of differently treated L360N steels in simulated solution.

Samples		Time (Day)					
		1	3	5	7	10	14
Base metal	Sterile	−0.73	−0.72	−0.71	−0.70	−0.70	−0.69
	SRB	−0.76	−0.79	−0.80	−0.81	−0.82	−0.83
IGHAZ	Sterile	−0.75	−0.74	−0.73	−0.72	−0.71	−0.70
	SRB	−0.78	−0.80	−0.82	−0.83	−0.84	−0.85
FGHAZ	Sterile	−0.76	−0.75	−0.74	−0.73	−0.72	−0.71
	SRB	−0.89	−0.86	−0.83	−0.84	−0.85	−0.86
CGHAZ	Sterile	−0.78	−0.87	−0.86	−0.85	−0.84	−0.83
	SRB	−0.81	−0.83	−0.85	−0.86	−0.83	−0.88

**Table 5:** EDS analysis results of surface corrosion products on the steel after 14 days of immersion in the sterile solution (wt.%).

Samples	C	O	Na	Si	Cl	Ca	P	Fe
Base metal	8.48	5.45		3.82	0.65	0.53	2.03	79.04
IGHAZ	7.07	7.32	4.46	-	-	-	3.12	78.03
FGHAZ	5.78	5.80	3.09	-	-	-	2.09	83.24
CGHAZ	7.06	12.12	1.73		2.22		2.34	74.53

**Table 6:** EDS analysis results of surface corrosion products on the steel after 14 days of immersion in the SRB-inoculated solution (wt.%).

Samples	C	O	Na	Si	Cl	Ca	P	Fe
Base metal	7.71	13.99	-	1.04	2.47	5.78	0.71	68.30
IGHAZ	4.28	8.73	-	-	3.41	2.09	-	81.49
FGHAZ	6.87	11.17	-	-	4.35	2.11	-	75.49
CGHAZ	12.41	14.79	2.85	0.42	5.43	10.31	1.36	52.42

## Data Availability

The original contributions presented in this study are included in the article/supplementary material. Further inquiries can be directed to the corresponding author.

## Supplementary Materials

The following supporting information can be downloaded at https://www.mdpi.com/article/10.3390/ma18184255/s1: Table S1: Fitted EIS parameters of BM in sterile solution during 14 days of testing; Table S2: Fitted EIS parameters of IGHAZ in sterile solution during 14 days of testing; Table S3: Fitted EIS parameters of FGHAZ in sterile solution during 14 days of testing; Table S4: Fitted EIS parameters of CGHAZ in sterile solution during 14 days of testing; Table S5: Fitted EIS parameters of BM in SRB-inoculated solution during 14 days of testing; Table S6: Fitted EIS parameters of IGHAZ in SRB-inoculated solution during 14 days of testing; Table S7: Fitted EIS parameters of FGHAZ in SRB-inoculated solution during 14 days of testing; Table S8: Fitted EIS parameters of CGHAZ in SRB-inoculated solution during 14 days of testing.
