# Supplementary material for: Effects of Multiple Welding Thermal Cycles on Stress Corrosion of L360N Steel in a Simulated Shale Gas Gathering Environment Containing Sulfate-Reducing Bacteria
Source: Materials (Basel). 2025 Sep 11;18(18):4255. doi: 10.3390/ma18184255 (PMC12471372; doi:10.3390/ma18184255)
Supplement: Supplementary file 1 [file materials-18-04255-s001.zip › materials-3761798-supplementary.pdf]

Supporting materials

Effects of Multiple Welding Thermal Cycles on Stress Corrosion of L360N Steel in a Simulated Shale Gas Gathering Environment Containing Sulfate-Reducing Bacteria

Table S1. Fitted EIS parameters of BM in sterile solution during 14 days of testing.

Time/d	R_s	Q_f		R_f	Q_f		R_{ct}
	/Ω·cm²	/Y_0 / S s^n·cm^{-2}	n	/Ω·cm²	/Y_0 / S s^n·cm^{-2}	n	/Ω·cm²
1	11.16	7.6×10^{-4}	0.68	66.7	2.1×10^{-3}	0.48	3499
3	9.56	2.1×10^{-3}	0.43	3.3	1.7×10^{-4}	0.80	7462
5	11.96	1.4×10^{-3}	0.88	264.9	1.8×10^{-3}	0.31	5816
7	10.64	1.8×10^{-2}	0.95	37.6	1.4×10^{-2}	0.92	7801
10	9.661	2.8×10^{-2}	0.91	19.19	1.5×10^{-2}	0.95	10320
14	10.08	2.1×10^{-2}	0.91	38.6	1.5×10^{-2}	0.91	9159

Table S2. Fitted EIS parameters of IGHAZ in sterile solution during 14 days of testing.

Time/d	R_s	Q_f		R_f	Q_f		R_{ct}
	/Ω·cm²	/Y_0 / S s^n·cm^{-2}	n	/Ω·cm²	/Y_0 / S s^n·cm^{-2}	n	/Ω·cm²
1	8.235	6.8×10^{-4}	0.95	2.7	1.4×10^{-4}	0.86	2463
3	8.993	1×10^{-2}	0.93	4.76	1.2×10^{-2}	0.97	4565
5	11.99	1.8×10^{-4}	0.85	630	1×10^{-3}	0.51	4500
7	11.95	1.8×10^{-3}	0.86	646.8	3.6×10^{-3}	0.47	5517
10	9.67	1.8×10^{-2}	0.94	3.16	1.7×10^{-2}	0.93	9334
14	12	1.2×10^{-3}	0.73	1010	3.6×10^{-3}	0.66	2230

Table S3. Fitted EIS parameters of FGHAZ in sterile solution during 14 days of testing.

Time/d	R_s	Q_f		R_f	Q_f		R_{ct}
	/Ω·cm²	/Y_0 / S s^n·cm^{-2}	n	/Ω·cm²	/Y_0 / S s^n·cm^{-2}	n	/Ω·cm²
1	12.05	9.8×10^{-3}	0.86	4.49	6.2×10^{-3}	1	2460
3	9.804	1.9×10^{-2}	0.9	8.1	8.4×10^{-3}	1	3021
5	10.5	2.2×10^{-2}	0.92	9.3	1.3×10^{-3}	0.92	3406
7	12.14	2×10^{-4}	0.85	790	6.3×10^{-4}	0.6	3916
10	11.1	2.5×10^{-4}	0.84	1013	5.4×10^{-4}	0.64	3754
14	10.83	4.2×10^{-4}	0.74	760.5	8×10^{-4}	0.5	1413

Table S4. Fitted EIS parameters of CGHAZ in sterile solution during 14 days of testing.

Time/d	R_s	Q_f		R_f	Q_f		R_{ct}
	/Ω·cm²	/Y_0 / S s^n·cm^{-2}	n	/Ω·cm²	/Y_0 / S s^n·cm^{-2}	n	/Ω·cm²
1	11.9	1.3×10^{-3}	0.85	400.9	1.6×10^{-3}	0.74	657.6
3	11.99	1.6×10^{-2}	0.85	5.8	9.6×10^{-3}	0.99	2648
5	10.16	1.9×10^{-3}	0.54	200.1	8.2×10^{-3}	0.87	2683
7	10.72	1.7×10^{-4}	0.84	1154	5.2×10^{-4}	0.75	1652
10	11.2	2.2×10^{-3}	0.9	929.1	5.8×10^{-3}	0.58	3612
14	11	1.3×10^{-2}	0.69	11.36	1.8×10^{-2}	0.81	127.9

Table S5. Fitted EIS parameters of BM in SRB-inoculated solution during 14 days of testing.

Time/d	R_s	Q_f		R_f	Q_f		R_{ct}
	/Ω·cm²	/Y_0 / S s^n·cm^{-2}	n	/Ω·cm²	/Y_0 / S s^n·cm^{-2}	n	/Ω·cm²
1	9.36	5.29×10^{-3}	0.9	2.6	1×10^{-2}	0.96	3045
3	9.35	1×10^{-3}	0.34	2.17	1.2×10^{-3}	0.88	2587
5	10.84	1.9×10^{-2}	0.92	8.5	5.5×10^{-3}	1	5589
7	10.46	2.5×10^{-2}	0.9	14.46	1.4×10^{-2}	0.94	5553
10	9.875	1.7×10^{-4}	0.88	96.01	3.9×10^{-4}	0.73	1848
14	11.55	1.1×10^{-2}	0.46	41.79	1×10^{-2}	1	3231

Table S6. Fitted EIS parameters of IGHAZ in SRB-inoculated solution during 14 days of testing.

Time/d	R_s	Q_f		R_f	Q_f		R_{ct}
	/Ω·cm²	/Y_0 / S s^n·cm^{-2}	n	/Ω·cm²	/Y_0 / S s^n·cm^{-2}	n	/Ω·cm²
1	9.562	1.3×10^{-2}	0.93	4.65	1×10^{-2}	0.92	2484
3	2.399	1.2×10^{-3}	0.4	9.8	4.7×10^{-4}	0.79	2085
5	13.03	1.5×10^{-2}	0.87	5.2	2.7×10^{-2}	0.9	3652
7	11.73	1.5×10^{-2}	0.73	274.5	3.6×10^{-3}	0.65	3954
10	10.6	5.3×10^{-4}	0.72	904	1.7×10^{-3}	0.50	1120
14	10	1.7×10^{-4}	0.87	958	4.2×10^{-4}	0.76	1635

Table S7. Fitted EIS parameters of FGHAZ in SRB-inoculated solution during 14 days of testing.

Time/d	R_s	Q_f		R_f	Q_f		R_{ct}
	/Ω·cm²	/Y_0 / S s^n·cm^{-2}	n	/Ω·cm²	/Y_0 / S s^n·cm^{-2}	n	/Ω·cm²
1	11.0	6.6×10^{-4}	0.79	180.4	2.4×10^{-3}	0.47	436.3
3	10.58	1.6×10^{-4}	0.85	697.2	1×10^{-3}	0.46	1981
5	10.69	3.1×10^{-4}	0.79	1358	9.2×10^{-4}	0.69	1399
7	12.24	1.1×10^{-3}	0.45	27.42	7.6×10^{-2}	0.98	2285
10	10.37	1.2×10^{-3}	0.64	8.5	1.4×10^{-4}	0.96	865.6
14	11.35	2.5×10^{-3}	0.87	1031	1×10^{-2}	0.76	1258

Table S8. Fitted EIS parameters of CGHAZ in SRB-inoculated solution during 14 days of

testing.

Time/d	R_s /Ω·cm²	Q_f /Y_0 / S s^n·cm^{-2}	n	R_f /Ω·cm²	Q_f /Y_0 / S s^n·cm^{-2}	n	R_{ct} /Ω·cm²
1	11.86	4.2×10^{-4}	0.78	244.7	2.8×10^{-3}	0.78	232.7
3	6.539	9.9×10^{-4}	0.24	508	1.5×10^{-4}	0.87	1132
5	11.3	5.9×10^{-4}	0.75	682.6	2.9×10^{-3}	0.5	1356
7	11.43	5.7×10^{-4}	0.73	723.8	1.8×10^{-3}	0.43	1375
10	11.73	5.7×10^{-3}	0.87	706	4.4×10^{-2}	0.89	185.7
14	10.76	3.5×10^{-4}	0.847	537.04	2.4×10^{-3}	0.88	33.91
